# Probing Single Synapses *via* the Photolytic Release of Neurotransmitters

**DOI:** 10.3389/fnsyn.2019.00019

**Published:** 2019-07-12

**Authors:** Diana E. Mitchell, Éric Martineau, Sabrina Tazerart, Roberto Araya

**Affiliations:** ^1^Department of Neurosciences, Faculty of Medicine, University of Montreal, Montreal, QC, Canada; ^2^The CHU Sainte-Justine Research Center, Montreal, QC, Canada

**Keywords:** dendritic spines, pyramidal neuron, non-linear microscopy, synaptic transmission, neocortex, two-photon (2P) uncaging

## Abstract

The development of two-photon microscopy has revolutionized our understanding of how synapses are formed and how they transform synaptic inputs in dendritic spines—tiny protrusions that cover the dendrites of pyramidal neurons that receive most excitatory synaptic information in the brain. These discoveries have led us to better comprehend the neuronal computations that take place at the level of dendritic spines as well as within neuronal circuits with unprecedented resolution. Here, we describe a method that uses a two-photon (2P) microscope and 2P uncaging of caged neurotransmitters for the activation of single and multiple spines in the dendrites of cortical pyramidal neurons. In addition, we propose a cost-effective description of the components necessary for the construction of a one laser source-2P microscope capable of nearly simultaneous 2P uncaging of neurotransmitters and 2P calcium imaging of the activated spines and nearby dendrites. We provide a brief overview on how the use of these techniques have helped researchers in the last 15 years unravel the function of spines in: (a) information processing; (b) storage; and (c) integration of excitatory synaptic inputs.

## Introduction

A major goal in neuroscience is to understand how neurons integrate and store information they receive from their synaptic inputs and, in turn, transmit signals to their postsynaptic targets. The pyramidal neuron, the most abundant in the cerebral cortex, is marked by a single axon (emanating from the soma in a straight fashion for the first 50–100 μm after it extensively ramifies), a long apical dendrite, several basal dendrites, and a pyramidal shaped soma (Spruston, 2008; Araya, 2016). Cortical pyramidal neurons are characterized by dendrites covered with dendritic spines—tiny protrusions along the dendritic tree which receive the majority (~95%) of excitatory inputs (Gray, 1959; Colonnier, 1968; Arellano et al., 2007). Dendritic spines have a very small head (~1 μm in diameter and <1 fL volume) and are separated from the dendrite by a slender neck (Arellano et al., 2007; Araya, 2014). Although these structures are incredibly small, the development of two-photon (2P) microscopy—which provides depth penetration in live tissue and 2P absorption only in the focal plane (Denk et al., 1990; Denk and Svoboda, 1997; Zipfel et al., 2003; Helmchen and Denk, 2005), and of 2P uncaging microscopy (Matsuzaki et al., 2001; Araya et al., 2006a)—where caged-neurotransmitters can be released from its cage only in the focal plane to mimic presynaptic release at single synapses—have allowed us to image and directly probe dendritic spine function in excitatory and inhibitory synaptic input transformations in pyramidal neurons (Matsuzaki et al., 2001; Araya et al., 2006a,b, 2007, 2014; Bloodgood and Sabatini, 2007; Tanaka et al., 2008; Harnett et al., 2012; Chiu et al., 2013; Oh et al., 2016). Importantly, probing the function of individual spines was not previously possible using standard electrophysiological techniques.

The uncaging of caged-compounds relies on converting an inert compound into its active form using light, by shattering the covalent bond connecting the aromatic cage and the neurotransmitter (Shoham et al., 2005). Two-photon uncaging of caged-compounds has become a widely used technique to optically manipulate single synapses (Matsuzaki et al., 2001; Araya et al., 2006a; Araya, 2014) and neuronal circuits (Nikolenko et al., 2007; Kim et al., 2016). Several caged neurotransmitters with relatively high two-photon absorption cross section have been developed (Ellis-Davies, 2007, 2019; Fino et al., 2009; Araya et al., 2013). Among those, nitrophenyl-, nitrobenzyl- and ruthenium-based caged neurotransmitters are the most successfully used in neuroscience to probe synapses and neuronal networks. In particular the development of caged-glutamate, with the use of either functionalized nitrobenzyl derivatives, such as 4-methoxy-7-nitroindolinyl-caged (MNI) glutamate (Canepari et al., 2001; Matsuzaki et al., 2001), or the use of a ruthenium polypyridine cage complex, such as Ruthenium-bipyridine-trimethylphosphine caged (RuBi) glutamate (Zayat et al., 2003, 2006; Fino et al., 2009), has proven to be a very powerful and effective way to photorelease glutamate in single dendritic spines (Matsuzaki et al., 2001; Fino et al., 2009; Araya, 2014; Tazerart et al., 2019). This technique has allowed us to study glutamatergic synaptic input transformations by dendritic spines in the dendrites of pyramidal neurons (Matsuzaki et al., 2001, 2004; Araya et al., 2006a,b, 2007, 2014; Nikolenko et al., 2008; Fino et al., 2009; Harnett et al., 2012; Tazerart et al., 2019).

Combining 2P uncaging of caged glutamate in single spines with 2P imaging of spine calcium (Bloodgood and Sabatini, 2005, 2007; Araya et al., 2006b; Chalifoux and Carter, 2010; Harnett et al., 2012; Beaulieu-Laroche and Harnett, 2018; Tazerart et al., 2019), voltage (Kwon et al., 2017), or the use of FRET-based sensors (Colgan and Yasuda, 2014; Nishiyama and Yasuda, 2015) is a powerful technique to probe the electrical (Araya et al., 2006b, 2014; Harnett et al., 2012; Tønnesen et al., 2014; Beaulieu-Laroche and Harnett, 2018) and biochemical processes at the level of a single synapse during synaptic transmission and plasticity (Araya, 2014; Colgan and Yasuda, 2014). Notably, calcium is an important signal for cellular processes, such as synaptic plasticity (Lynch et al., 1983; Malenka et al., 1988; Artola and Singer, 1993; Cummings et al., 1996; Fino et al., 2010). It has been shown that local concentration differences in dendrites and spines are associated with the induction of long-term plasticity (LTP, high calcium concentration) or long-term depression (LTD, low calcium concentration; Lisman, 1989; Ismailov et al., 2004; Nevian and Sakmann, 2006). A widespread approach for combining 2P uncaging of caged neurotransmitters with 2P imaging (e.g., calcium) in the activated spines and nearby dendrites has been to use two pulsed-lasers (Matsuzaki et al., 2004; Bloodgood and Sabatini, 2005): one laser for 2P uncaging of caged neurotransmitters (i.e., MNI-glutamate using 720 nm excitation light), and a second laser for 2P excitation of calcium indicators in the activated spine (s) (i.e., Fluo-4 using 800–850 nm excitation light). This configuration allows for the simultaneous uncaging of caged neurotransmitters and calcium imaging of events in single spines (and/or imaging the short- or long-term changes in the morphology of spines loaded with other fluorophores) during development and during the induction of synaptic plasticity (Matsuzaki et al., 2004; Harvey and Svoboda, 2007; Lee et al., 2016). In addition, the two 2P laser configuration has been widely used to simultaneously uncage in single spines and image FRET-based sensors (Colgan and Yasuda, 2014; Nishiyama and Yasuda, 2015; Tang and Yasuda, 2017). This approach, however, is costly and not a possibility for all laboratories, especially those just starting up.

Here, we describe a cost-effective description of the components necessary for the construction of a one laser source-2P microscope capable of nearly simultaneous 2P uncaging of neurotransmitters and 2P calcium imaging of the activated spines and nearby dendrites using a single wavelength with low-laser power for calcium imaging (power not sufficient to result in any partial uncaging of the caged glutamate) and short high-laser power pulses to uncage caged glutamate. In addition, we describe the anticipated results that can be obtained with this microscope configuration as well as an overview on how the 2P uncaging of caged glutamate to activate single dendritic spines has helped in understanding spine function in: (a) information processing; (b) storage; and (c) integration of excitatory synaptic inputs.

## Materials and Equipments

### Two Photon Set-up

**Figure 1:**
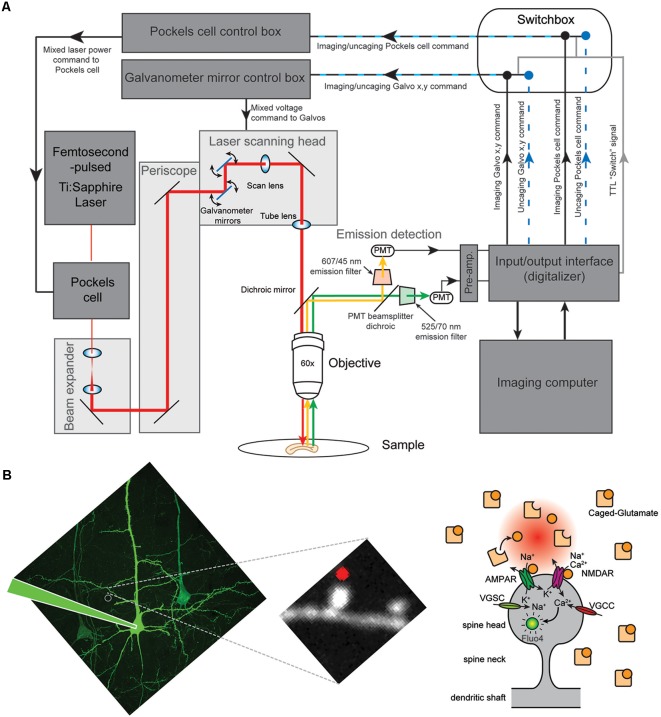
Two photon uncaging of caged glutamate at single spines of layer 5 (L5) pyramidal neurons. **(A)** Schematic illustration of the custom imaging system. A customized high-speed electronics module by Bruker (formerly Prairie Technologies) is integrated between the input/output interface and the laser scanning control boxes to rapidly switch (in less than 2 ms) between the command strings (imaging or photoactivation mode) being routed to the control boxes. The module is fully integrated with the imaging software and allows synchronized photoactivation (uncaging), imaging and electrophysiology. This gives the ability to set-up sophisticated experimental protocols in a user-friendly manner using a single laser source and scanning system. (**B**; *Left*) Two-photon (2P) image of a L5 pyramidal neuron from V1 cortex and a zoomed in image of a piece of dendrite showing the spines. (*Right*) Cartoon drawing of a dendritic spine. AMPAR, AMPA receptor; NMDAR, NMDA receptor; VGSC, voltage-gated sodium channel; VGCC, voltage-gated calcium channel.

- *Laser*: a femtosecond-pulsed Ti:Sapphire laser from Coherent Inc., Santa Clara, CA, USA was used for imaging and uncaging (see “Procedures” section for details). More specifically, we used the Chameleon ULTRA II ultrafast tunable Ti:Sapphire laser, which provides 140 femtosecond pulses of near infrared light (NIR) from 680 nm to 1,080 nm, that scatters less in tissue and induces less photodamage than shorter wavelengths, with a peak power of ~3.5 W at 800 nm. In particular, our laser has a Ti:Sapphire oscillator with a 80 MHz repetition rate. In addition, the tunable capabilities of the Ti:sapphire laser allow us to perform experiments in which different excitation wavelengths are needed (e.g., ~810 nm excitation light for fluorescent calcium indicators and 2P uncaging of RuBi-glutamate or ~720 nm for 2P uncaging of MNI-glutamate, see below) and have the freedom to excite a wide range of fluorophores.
- *Optical table*: laser light is delivered to the scan head and microscope (see below) through a series of optical elements that include a Pockels Cell (see below), mirrors, retardation wave plate (lambda/2), beam expander (set of lenses to act as a telescope to expand the laser beam) to change the beam size and overfill the back aperture of either a 60× and of a 40× microscope objective. The retardation wave plate is placed in the optical path for experiments where the polarization of the laser beam needs to be directed.
- *Pockels Cell*: for the experiments presented here, where fast and dynamic control of light intensity from pulsed femtosecond lasers with great contrast is needed, we used an electro-optical laser modulator (model 350–80 Pockels Cell), and driver (model 302RM driver, DC-to-250 KHz bandwidth, 1-microsec rise/fall time, 750 V. max. output) from ConOptics Inc., Danbury, CT USA. These devices allow us to control at high speed the intensity of light—e.g., fast change from high-laser-power-neurotransmitter-uncaging mode to a low-laser-power-spine-imaging mode. This laser modulator and its driver are extremely reliable devices that we have extensively used in the past (Araya et al., 2006a,b, 2007, 2013; Fino et al., 2009; Tazerart et al., 2019).
- *Scanning system*: a Bruker Inc., Billerica, MA, USA (formerly Prairie Technologies Inc.) scan head with a single pair of 6 mm galvanometer mirrors was mounted on an Olympus upright BX51WI microscope connected to a PC workstation unit with *PrairieView* software for frame scanning, line scanning, region of interest (ROI) selection, scan rotation and optical zoom modes. The scan box is optically linked to the Olympus microscope (Figure 1A). Importantly, this scanning system uses the same pair of galvanometer mirrors for both imaging and uncaging.
- *Objectives*: the light is focused using a high numerical aperture (NA) objective that confines the light spatially, while the pulsed-laser provides a concentration of photons in time. Specifically, we used a 60× 0.9 NA water immersion objectives from Olympus. In addition, a 10× (0.3 NA) objective to easily locate the neurons from different cortical layers and the patch pipettes for patch-clamp recordings was used.
- *Fluorescence detection*: high NA objectives (see “Objectives” section) are used to collect as many emission photons as possible from the sample—where two-photon (2P) absorption and excitation of fluorescence from the excited region of the sample irradiates fluorescence in all directions. A set of two top mounted external photomultiplier tube (PMT) detectors, controlled by a high voltage power supply, and designed to optimize collection efficiency when used with 40× and 60× microscope objective lenses were used. In particular, we used two Hamamatsu multi-alkali PMTs with low dark current (10 nA) and high sensitivity (8,500A/lumen) each with a specific emission filter (525/70 nm and 607/45 nm) and 575 nm dichroic beam splitter, allowing for the simultaneous viewing and acquisition from both detectors. The signal obtained from the PMTs is then directed to a pre-amplifier, which is then directed to the Prairie view acquisition board. *TriggerSync* software integrates the collection of PMT based fluorescence data with electrophysiology (see below). Multiple inputs and outputs are independently programmable for customized experimental protocols designed by the user. All data (PMT and electrical) are recorded by a single computer for accurate time synchrony. To block any reflected laser light into the PMT during each uncaging pulse we placed an IR filter before the PMT dichroic. Alternatively, fast shutters can be employed.
- *High-speed electronics module (“Switch box”)*: a customized high-speed electronics module by Bruker (formerly Prairie Technologies) that allows us to switch between imaging and photoactivation (uncaging) mode in less than 2 ms. The module is fully integrated with the *PrairieView* software and allows synchronized photoactivation (uncaging) and electrophysiology. The module consists of a number of 2 × 1 multiplexers to integrate and route imaging and uncaging commands to the galvanometer and Pockels Cell controllers as directed by the user at very precise time points. This gives us the ability to set up sophisticated experimental protocols in a user-friendly manner using a single laser two-photon scanning/uncaging system. This module receives: (1) the software-driven imaging and uncaging Galvanometer mirror (X-Y scanning) commands; (2) the imaging and uncaging laser power command (the voltage to the Pockels cells); and (3) the software-controlled TTL pulse, the “Switch” command (Figure 1A). In the absence of a TTL pulse, this module outputs the imaging scanning and laser power commands to the Galvanometer mirrors and Pockels cell control boxes, respectively. Upon reception of a TTL pulse (5V), the module rapidly switches to uncaging mode output (~2 ms delay) and sends the uncaging scanning and laser power commands to the same control boxes, effectively overriding the imaging commands for the duration of the TTL pulse. In essence, this module “switches” a single pair of Galvanometer mirrors and laser power source between an “imaging mode” and an “uncaging mode” by alternating which set of commands is output to the control boxes.

### Electrophysiology

MultiClamp 700 B amplifiers (Molecular Devices) were used for electrophysiological recordings in layer 5 (L5) pyramidal neurons with a patch electrode filled with internal solution (see “Solutions” section). *Patchstar* micromanipulators and software from Scientifica Inc., Coral Gables, FL, USA were used. A motorized movable X-Y base plate from Scientifica Inc., Coral Gables, FL, USA was used to place the micromanipulators and the brain slice chamber to move them independently of the microscope and light path. DIC optics were used to clearly visualize and patch the soma of pyramidal neurons. A camera and monitor were used to visualize the patch pipette and neurons for somatic patching.

### Mice

C57B/6 mice, obtained from Jackson Laboratory.

### Tissue

Coronal visual cortex brain slices.

### Caged Neurotransmitters

4-methoxy-7-nitroindolinyl (MNI)-caged L-glutamate (2.5 mM; Tocris) or Ruthenium-bipyridine-trimethylphosphine (RuBi)-caged glutamate (800 μM; Tocris) were used.

### Solutions

Artificial cerebrospinal fluid (ACSF), containing (in mM) 126 NaCl, 26 NaHCO_3_, 10 Dextrose, 1.15 NaH_2_PO_4_, 3 KCl, 2 CaCl_2_, 2MgSO_4_, Internal solution containing (in mM) 0.3 Fluo-4, 0.1 Alexa-568 (to use with MNI-caged glutamate) or Alexa-594 (to use with RuBi-caged glutamate), 130 Potassium D-Gluconic Acid (Potassium Gluconate), 2 MgCl_2_, 5 KCl, 10 HEPES, 2 MgATP, 0.3 NaGTP, pH 7.4, and 0.4% Biocytin; Sucrose cutting solution, containing (in mM) 27 NaHCO_3_, 1.5 NaH_2_PO_4_, 222 Sucrose, 2.6 KCl, 1 CaCl_2_, and 3 MgSO_4_.

### Note on the Selection of Caged-Compounds and Fluorophores

Since a single 2P laser source is used, one must select a caged neurotransmitter, that can be uncaged with short high-laser power pulses, and a fluorophore that is excited at the same wavelength but with a laser power that is not sufficient to uncage the caged neurotransmitter (Araya et al., 2006b; Tazerart et al., 2019). For instance, in the experiments presented here, where glutamate uncaging is performed to activate a single spine (with high laser power on sample) nearly simultaneously with imaging fluorescence in dye-loaded dendritic spines (with low laser power on sample) to uncover their morphology, MNI-glutamate and Alexa-568 or RuBi-glutamate and Alexa-594 were used at 720 or 810 nm, respectively. In experiments where 2P uncaging of caged glutamate onto single spines was paired with nearly simultaneous calcium imaging in the activated spines, we chose RuBi-glutamate uncaging in Alexa-594 and Fluo-4 loaded L5 pyramidal neurons using a wavelength of 810 nm. Another important consideration in choosing a calcium sensor for these experiments is its affinity for calcium, signal-to-noise ratio, dynamic range and response kinetics. We chose Fluo-4 to measure spine calcium signals due to its high dynamic range, its brightness (low power to excite), low Kd (345 nM), and high signal-to-noise ratio. Furthermore, it is a widely used indicator to measure spine calcium signals (Harnett et al., 2012; Araya et al., 2014; Beaulieu-Laroche and Harnett, 2018). In addition, Fluo-4 is one of the lowest Kd green indicators with a peak 2P excitation absorption in the 800 nm range (Svoboda and Yasuda, 2006). Other alternatives include Calcium Green-1 (Kd = 190 nM) or Fluo-8 (Kd = 389 nM).

## Procedure

### Brain Slices Preparation and Electrophysiology

Brain slices were made from C57B/6 mice, aged postnatal day 14–21 as described previously (Araya et al., 2006a,b, 2007, 2014). Brains were removed and submerged in cold (4°C) sucrose cutting solution saturated with 95% O_2_, 5% CO_2_. In these experiments, we prepared coronal slices of the visual cortex that were 300 μm thick. Slices were incubated in ACSF saturated with 95% O_2_, 5% CO_2_, at 32°C for 30 min and then at room temperature until ready for use (~40 min). Recordings were made from the soma of layer 5 (L5) pyramidal cells in the current-clamp configuration with MultiClamp 700B amplifiers (Molecular Devices) using a pipette (pulled from borosilicate glass tubes) filled with the internal solution described above (see “Materials and Equipments” section). The membrane potential of cells was held at −65 mV in current-clamp configuration throughout the recordings. We only used cells for which the injected current to hold the cell at −65 mV was <100 pA.

### Near-Simultaneous 2P Imaging and Uncaging of Dendritic Spines in Layer 5 (L5) Pyramidal Cells

Once a successful patch was obtained, cells were allowed to dye fill for ~25 min for visualization of spines located on the basal dendrites for high magnification imaging and uncaging. Then, we used the above-described 2P laser scanning microscope to acquire morphological images of dendritic spines of L5 pyramidal neurons. Excitation light of ~5–8 mW on sample was used (i.e., after the objective) at a wavelength of 720 nm in neurons filled with Alexa-568 or 810 nm in neurons loaded with Fluo-4 and Alexa-594. These images allowed us to identify dendritic spines of interest.

Once the neuron was allowed to dye-fill and morphological images were taken, MNI- or RuBi-caged glutamate (named from here on MNI-glutamate and RuBi-glutamate for simplicity) was added to the bath solution at a final concentration of 2.5 mM or 800 μM, respectively. Morphological images of selected spines were used to position the uncaging spot ~0.3 μm away from the edge of the spine head as previously described (Araya et al., 2006a,b, 2007, 2014). Uncaging of MNI- or RuBi-glutamate was performed using a wavelength of 720 nm or 810 nm, respectively, and a laser power of ~25–30 mW on sample for 4 ms. Uncaging-evoked excitatory postsynaptic potentials (uEPSPs) were recorded at the soma through the patch pipette. We have previously published control experiments showing the stability of uEPSP amplitude or spine morphology over time (~30 min; see Figure S2 from Tazerart et al., 2019). Importantly, 2P uncaging of glutamate in single spines with this uncaging protocol not only induces uEPSPs of similar amplitude to spontaneous (s) EPSPs, but also recapitulates the correlations observed between spine morphology (head size and neck length) and EPSP amplitude when single spines are activated with minimal electrical stimulation (see “Results” section and Araya et al., 2014).

Alternatively, high concentrations of caged-glutamate compounds (~10 mM MNI-glutamate) can be applied locally with a pipette positioned close to the selected spine and parent dendrite and uncaged using shorter laser pulses (<1 ms; Matsuzaki et al., 2001, 2004; Losonczy and Magee, 2006; Harnett et al., 2013; Beaulieu-Laroche and Harnett, 2018). Although this approach was not used in this study, it has the advantage of using shorter pulses, shortening the delay between sites for multi-site (spine) uncaging, and of producing uEPSPs with kinetics almost identical to spontaneous EPSPs (sEPSPs; see below). However, it cannot ensure that the added caged-compound concentration is stable and uniform across all uncaging sites. Altogether, uncaging parameters (laser power, pulse length and caged-compound concentration) can be adjusted according to the experimental needs of the user.

To uncage glutamate at a single spine while near-simultaneously imaging calcium signals (e.g., Fluo-4 loaded cells) and/or morphological changes (Alexa-568 or -594 loaded cells), imaging was performed for 500 ms before uncaging and almost immediately after 2P uncaging for at least 600 ms. This was achieved by sending a 7 ms TTL-pulse to the switchbox, starting 2 ms before the start of the 4 ms uncaging command. The duration of the TTL-pulse is determined based on the switchbox delay (<2 ms) and the duration of the uncaging pulse in order to ensure that the system will be in “uncaging mode” for the entire duration of the uncaging command (in our case, 2 ms before plus 1 ms after). Switching from imaging mode to uncaging mode effectively interrupts the imaging for ~7 ms, allowing 2P uncaging of caged-glutamate in the selected spine.

Two different imaging strategies were used based on our experimental needs, “linescan imaging” and “ROI imaging.” For experiments where a high temporal resolution is required, such as those designed to report and analyze fast spine calcium signals, a single line across the middle of the spine head was scanned at high speed (1.6 μs dwell time; ~1 ms/line). For experiments where more spatial information is required, such as those where the calcium signal of not only the activated spine head but also of the parent dendritic shaft and neighboring spines is required, as well as the activated spine morphological changes, the imaging acquisition was set to a small portion, or ROI, of the entire field of view (~150 × 150 pixels). Images were acquired at ~30 Hz, averaged eight times, with 8 μs dwell time (image period ~300 ms). For ROI imaging, the uncaging command and TTL “switch” pulse were set to occur in the lag period between two images to ensure that no image is interrupted while being scanned (no pixels are skipped) by the 2P uncaging pulse. In both cases, Alexa-594 fluorescence was acquired simultaneously with Fluo-4 fluorescence, serving both as a normalization signal for the analysis of calcium signals (see below).

### Analysis of Calcium Linescan Signals

Analysis of calcium linescans obtained during uncaging was performed using custom algorithms (MATLAB; MathWorks). We restricted this analysis to the portion of the linescan that overlapped with the spine head. The fluorescence of each line was calculated as the mean of all pixels. The relative change in calcium levels ($\frac{\Delta G}{R}$) over time was calculated using the following formula:

(1) $$\frac{\Delta G}{R}=\frac{G-G_{\text{baseline}}}{R}$$

where *G* is the fluorescence from the Fluo-4 dye and *R* is the fluorescence from the Alexa-594 dye. *G*_baseline_ is the mean Fluo-4 fluorescence of the first image of the sequence.

### Analysis of Calcium ROI Signals

Analysis of calcium images obtained during uncaging was performed using custom algorithms (MATLAB; MathWorks). We confined the quantification of the change in Fluo-4 fluorescence to either the spine head or the dendrite. We manually drew ROIs in the shape of a circle for spine heads and of a polygon for dendrites. The fluorescence of each image was calculated as the mean of all pixels within each ROI. The relative change in calcium levels ($\frac{\Delta G}{R}$) over time was calculated using equation 1. To show the change in calcium signal in the activated spine and parent dendrite, we calculated the change in Fluo-4 fluorescence (Δ*F*) on the ROI images obtained, from the first image in the sequence.

### Ethics

This study was performed in compliance with experimental protocols (13-185, 15-002, 16-011, 17-012, 18-011 and 19-018) approved by the *Comité de déontologie de l’expérimentation sur les animaux* (CDEA) of the University of Montreal.

## Results

### Activation of Single Dendritic Spines Using Two-Photon (2P) Uncaging of Caged Glutamate

**Figure 2:**
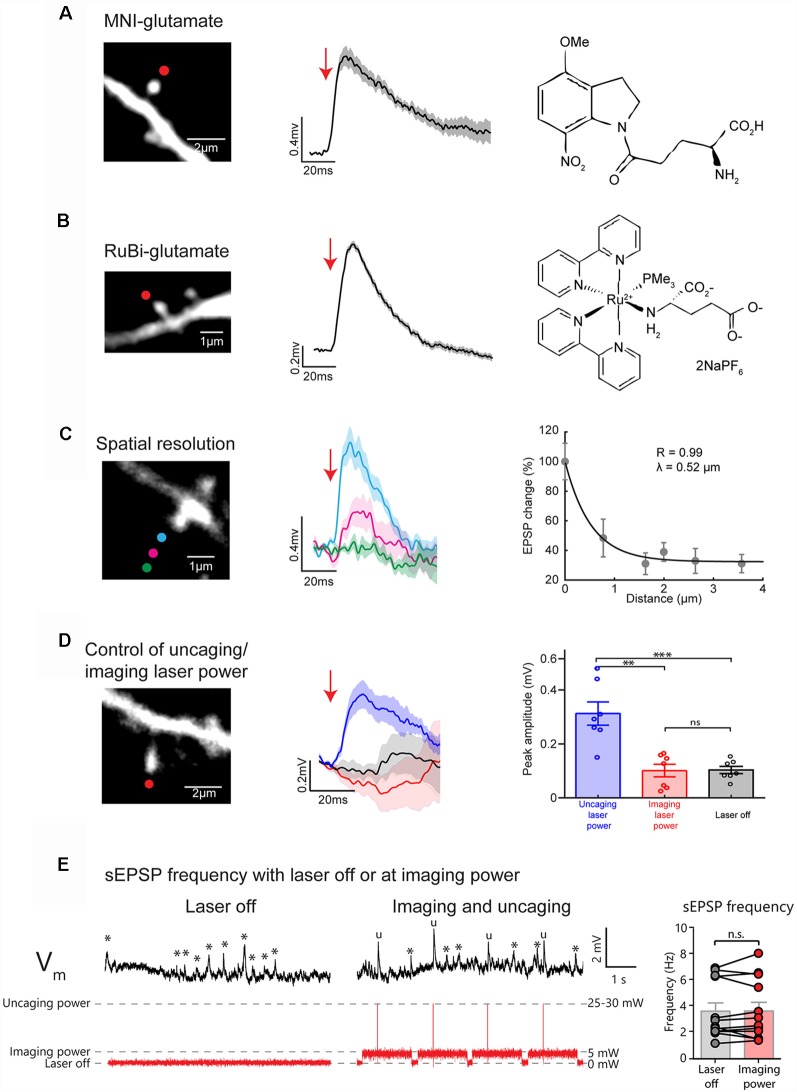
Uncaging caged glutamate at single spines evokes uncaging (u)EPSPs detected at the soma of layer 5 (L5) pyramidal neurons with high spatial precision. **(A)** Representative 2P uncaging of MNI-glutamate experiment in a single spine. (*Left*) Two-photon (2P) image of a single spine in the basal dendrites of a L5 pyramidal neuron (red dot indicate site of uncaging). (*Center*), 2P uncaging of caged MNI-glutamate near the selected spine induces a uncaging-evoked excitatory postsynaptic potential (uEPSP) measured at the soma in current-clamp configuration. The tick line and the shaded area represent the mean ± standard error of the mean (SEM) of 10 uEPSP generated at this spine. (*Right)* Chemical formula for MNI-glutamate. **(B)** Representative 2P uncaging of RuBi-glutamate experiment in a single spine. (*Left*), 2P image of a single spine on the basal dendrites of a L5 pyramidal neuron (red dot indicate site of uncaging). (*Center*) 2P uncaging of caged RuBi-glutamate near the selected spine induces a uEPSP measured at the soma in current-clamp configuration. The tick line and the shaded area represent the mean ± SEM of 10 uEPSPs generated at this spine. (*Right*) Chemical formula for RuBi-Glutamate. **(C)** Representative 2P uncaging of MNI-glutamate in a single spine spatial resolution experiment. (*Left*) 2P image of a single spine in the basal dendrites of a L5 pyramidal neuron with colored circles showing the location of different MNI-glutamate uncaging spots. (*Center*) 2P uncaging in the selected spine at different locations and their corresponding uEPSPs measured at the soma. The tick line and the shaded area represent the mean ± SEM of 10 uEPSPs generated at this spine. (*Right*) Relationship between the distance of the uncaging spot from the spine and the evoked uEPSP. Each dot corresponds to the mean ± SEM of 10 uEPSPs generated a given distance from a single spine. **(D)** Representative experiment to explore the effect of 2P uncaging pulses in a single spine with imaging or uncaging power. (*Left*) 2P image of a single spine on the basal dendrites of a L5 pyramidal neuron showing the uncaging spot (red dot). (*Center*) 2P uncaging of RuBi-glutamate right next to the selected spine using uncaging (blue trace) or imaging laser power (red trace) while uEPSP are recorded at the soma. Note the absence of induction of a uEPSPs when short pulses at imaging laser power were given. Black traces represent the averaged membrane potential recorded (10 trials) while the laser was off, 100 ms before the onset of uncaging laser pulses. Thicker traces are an average of 10 uEPSPs, and shaded areas illustrate ± SEM. (*Right*) Plot showing the peak amplitude (mV) of the responses measured in seven independent experiments, from *n* = 7 independent spines, where the average triggerd 2P uEPSP (10 trials per spine) was recorded at either uncaging (blue bar and points) imaging laser power (red bar and dots), or when the laser was off (black bar and dots). **p < 0.01; ***p < 0.001; *ns*, not significant. **(E)** Example traces of membrane voltage recorded at the soma of a L5 pyramidal neuron while the laser was off (*Left*) or at imaging laser power during an uncaging/imaging protocol (*Center*). Asterisks indicate the presence of a spontaneous EPSPs (sEPSPs) while “u” marks uEPSPs. Note the similar frequency of sEPSPs (*) when the laser is off or at imaging laser power. (*Right*) Plot represents the frequency of events measured over a 30–60 s period before and during the uncaging/imaging protocol for each spine (*n* = 12 cells). *ns*, not significant. The first 50 ms following uncaging pulses were not included in this analysis.

Two photon (2P) glutamate uncaging is an effective tool to locally release caged glutamate and activate glutamate receptors at a precise dendritic location to evoke a depolarization (Figure 1B) that can mimic the physiological stimulation of a single synapse (Matsuzaki et al., 2001, 2004; Araya et al., 2006a,b, 2007, 2013, 2014; Fino et al., 2009; Tazerart et al., 2019). Here, we performed 2P uncaging of bath applied MNI-glutamate (2.5 mM; Figure 2A) or RuBi-glutamate (800 μM; Figures 2B,C)—two commercially available caged glutamate compounds with relatively high two-photon absorption cross section (Canepari et al., 2001; Matsuzaki et al., 2001; Zayat et al., 2003, 2006; Fino et al., 2009)—at a single spine on basal dendrites of L5 pyramidal neurons from mouse V1 cortical slices. Short laser pulses (4 ms, ~25–30 mW on sample) just above the spine head (Figures 2A,B) were delivered while recording the uncaging-evoked excitatory postsynaptic potentials [uncaging(u)EPSP] at the soma using whole-cell recordings in current-clamp mode. Two-photon uncaging MNI-glutamate or RuBi-glutamate induced a sharp depolarization that is similar in amplitude (MNI-glutamate: 0.65 ± 0.06 mV; RuBi-glutamate: 0.74 ± 0.011 mV) and that has a slightly slower rise time and slightly longer duration (10/90 rate of rise: 0.063 ± 0.01 and 0.056 ± 0.02 mV/ms; duration: 124.6 ± 17.1 and 108.14 ± 13.25 ms, for uncaging MNI-glutamate and RuBi-glutamate, respectively) than sEPSPs (Figures 2A,B; Fino et al., 2009; Araya et al., 2014; sEPSP amplitude: 0.86 ± 0.07 mV; sEPSP 10/90 rate of rise: 0.25 ± 0.03 mV/ms; sEPSP duration: 50.4 ± 4 ms; Araya et al., 2006b).

A main feature of 2P glutamate uncaging is that it allows for the precise activation of a single synapse (Matsuzaki et al., 2001; Araya et al., 2006a,b; Fino et al., 2009). To test the 2P uncaging spatial resolution of our approach, we targeted the laser at six different locations that were located at a range of distances from the head of the spine (the first three locations are shown in Figure 2C). Importantly, uncaging at locations further from the spine head generated smaller uEPSPs (Figure 2C). To quantify the spatial resolution of uncaging, we plotted the uEPSP amplitude as a function of distance of the uncaging spot from the spine head. We fit these data with an exponential curve, which revealed a distance constant (λ) of ~0.5 μm (Figure 2C). Hence, with this experimental configuration, we have single-spine uncaging spatial resolution, which allowed us to rapidly and precisely mimic synaptic activation of single spines while synchronously recording the uEPSP at the soma, as we have shown before (Araya et al., 2006a,b).

Another important technical control is to ensure that the imaging laser power is not sufficient for the photolytic release caged-glutamate. We previously showed that 5–8 mW at 720 nm is insufficient to uncage MNI-glutamate (for detailed control experiments see Figure S9 from Tazerart et al., 2019). We now show that short laser pulses of 5–8 mW at 810 nm are not sufficient to uncage RuBi-glutamate or to induce any postsynaptic response, while short pulses of 25–30 mW on sample reliably induced uEPSPs at the same dendritic spines (Figure 2D; 0.31 ± 0.043 mV vs. 0.101 ± 0.023 mV, *n* = 7 spines, Paired *t*-test, *p* = 0.004). To further validate that imaging laser power does not inadvertently uncage RuBi-glutamate, we performed experiments where we looked at the frequency of sEPSPs during periods where the laser was off or on at imaging power while scanning a dendritic spine (Figure 2E). These results showed that the frequency of sEPSP in each cell was similar during periods where the laser was off vs. at imaging laser power (Figure 2E; Laser Off: 3.63 ± 0.64 Hz, Imaging laser power: 3.65 ± 0.67 Hz; *n* = 12 spines; Paired *t-test*, *p* = 0.936).

### Nearly Simultaneous 2P Calcium Imaging and 2P Uncaging of Caged Neurotransmitters With One Pulsed-Laser Source

Calcium is an important signal for cellular processes, such as the induction of LTP or LTD (Lynch et al., 1983; Malenka et al., 1988; Lisman, 1989; Artola and Singer, 1993; Cummings et al., 1996; Ismailov et al., 2004; Nevian and Sakmann, 2006; Fino et al., 2010). Since 2P uncaging of MNI-glutamate or RuBi-glutamate has excellent spatiotemporal resolution (Figure 2C, and see Araya et al., 2006b, 2007; Fino et al., 2009), combining 2P calcium imaging with 2P uncaging of neurotransmitters is a powerful approach to probe the mechanism of cellular processes (e.g., LTP or LTD) at the level of a single synapse. To demonstrate that a single laser configuration is suitable to perform such an approach, we performed near-simultaneously 2P glutamate uncaging and calcium-imaging at the activated spines in the basal dendrites of L5 pyramidal neurons.

**Figure 3:**
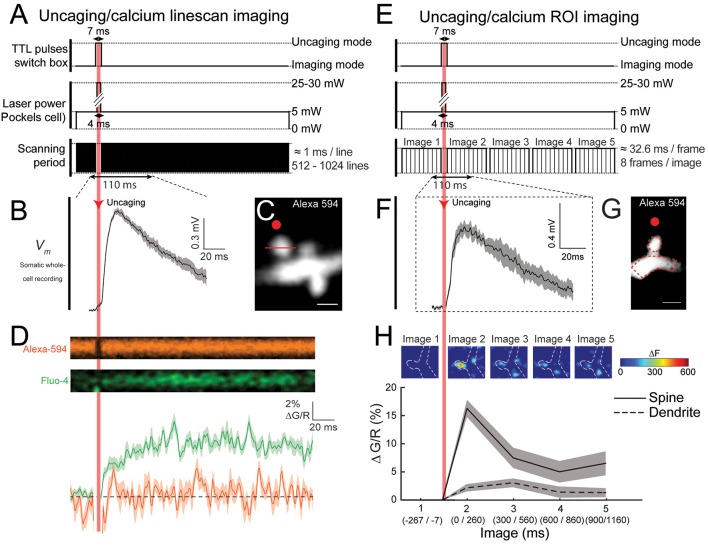
Near simultaneously imaging of calcium dynamics in the spines of layer 5 (L5) pyramidal neurons following glutamate uncaging. **(A)** Schematic representation of the TTL pulses being sent to the switchbox, the change in laser power over time and the imaging scanning period during a single trial, where each black line represents the beginning of a new linescan. Note the very brief interruption in the imaging during the uncaging. **(B)** Averaged uEPSP, recorded at the soma, evoked by 2P glutamate uncaging targeted 0.3 μm away from the head of the selected spine (20 trials). **(C)** 2P image of the selected spine of a L5 pyramidal neuron. Red dot: the uncaging spot; Red line: position of the linescan during calcium signal acquisition. **(D)** Top panel: linescan images illustrating the change in Alexa-594 and Fluo-4 fluorescence over time during a single trial. Bottom panel: quantification of the average change in the calcium and Alexa-594 signal ($\frac{\Delta G}{R}$) in the spine head following 2P glutamate uncaging for 10 trials. **(E)** Schematic representation of the TTL pulses being sent to the switchbox, the change in laser power over time and the scanning period during a single trial, where each thin black line represents the beginning of a new framescan and each black box represents a single averaged image being generated. **(F)** Averaged uEPSP, recorded at the soma, evoked by glutamate uncaging targeted 0.3 μm away from the head of the selected spine (20 trials). **(G)** 2P image of the selected spine of a L5 pyramidal neuron. The red dot represents the uncaging spot. **(H)** Top: color-coded images of the changes in fluorescence across the region of interest (ROI) during a single trial (Δ*F*). Quantification of the average change in the calcium signal ($\frac{\Delta G}{R}$) in the spine head and parent dendrites following glutamate uncaging at the spine head for 20 trials.

First, we used a “linescan” approach to track calcium signals in the activated spine head with high spatiotemporal precision (Figures 3A–D). Briefly, a line through the middle of the spine located in the basal dendrites of L5 pyramidal neurons is scanned before and after 2P uncaging of caged glutamate at a spot positioned ~0.3 μm from the spine head (Figure 3C, line and red spot, respectively). As shown in Figure 3B, 2P glutamate uncaging reliably induced somatic uEPSPs (average of 10 depolarizations, amplitude: 0.99 ± 0.03 mV; duration: 185.2 ± 23.8 ms; ranging from ~0.81 to 1.14 mV in amplitude, and from 100.6 to 292.4 ms in duration), and an increase in spine calcium signals in the activated spine head of Fluo-4 loaded neurons. Importantly, the fluorescence from the Alexa-594 dye remained constant before and after the 2P uncaging of glutamate (Figure 3D), showing that the application of a short, high power, laser pulse next to the spine does not damage the spine head, which otherwise would trigger fluctuations in Alexa-594 fluorescence after the uncaging pulse. We quantified these calcium dynamics using a custom algorithm in MATLAB by calculating the ratiometric measurement $\frac{\Delta G}{R}$ (see “Procedures” section). This method of quantification allows us to measure calcium signals that are insensitive to small fluctuations in basal calcium levels and independent of the spine head volume (Sabatini et al., 2002; Bloodgood and Sabatini, 2007). The trace in the lower panel of Figure 3D shows the average change in fluorescence from baseline following 10 uncaging events at this dendritic spine. Initially, calcium levels are quite stable with a rapid increase immediately following 2P uncaging of glutamate right next to the activated spine head. We observed a peak calcium single of 12.0% ± 0.54% $\frac{\Delta G}{R}$ (Figure 3D). Using this approach, we observed a range of calcium signals in the activated spine heads between 3% and 20% $\frac{\Delta G}{R}$, which is consistent with previous published results obtained using a similar analytical method (Sabatini et al., 2002; Yasuda et al., 2003; Bloodgood and Sabatini, 2007).

Next, we used an “ROI-scan” approach to monitor the spatial dynamics of calcium signaling in the activated spines, parent dendrite and neighboring spines (Figures 3E–H). In the example presented in Figures 3E–H, a small portion of the entire field of view of the ROI containing the spine of interest and its parent dendrite are imaged before and after 2P uncaging of caged glutamate at a spot positioned ~0.3 μm away from the spine head (Figure 3G, red spot) is performed. The activation of a single spine by 2P uncaging of glutamate (RuBi-glutamate) reliably induced somatic uEPSPs (Figure 3F, average of 20 depolarizations), and clear calcium signals in the activated spine head, which remains elevated for ~300 ms (Figure 3H, image 2) before decreasing back to baseline levels (Figure 3H, insets). Interestingly, the images depicted in Figure 3H show that calcium increases (Δ*F*) remained mostly located in the spine head following its activation and a much smaller response occurred in the parent dendrite (Figure 3H, insets). Using custom algorithms in MATLAB, which measures the change in fluorescence ($\frac{\Delta G}{R}$) over time within a selected area (i.e., spine head or dendrite), these calcium dynamics can be further quantified (see “Procedures” section). Calculation of $\frac{\Delta G}{R}$ clearly shows that calcium signals were initially minimal in the spine head (image 1 in Figure 3H insets), whereas following uncaging they reach levels 16.2% ± 1.6% $\frac{\Delta G}{R}$ (image 2 in Figure 3H, insets). Interestingly, calcium signals were considerably smaller in the parent dendrite, only reaching values of ~5% $\frac{\Delta G}{R}$ (Figure 3H), revealing that dendritic spines favor calcium level rises following synaptic activation (Figure 3H)—acting as biochemical compartments that can effectively confine calcium in the spine head without affecting neighboring spines, a pretty well established function of spines (Araya, 2014). Hence, depending on experimental requirements (high spatial vs. high temporal resolution), either a “linescan” or an “ROI-scan” approach can be used to track calcium dynamics in spines after fast 2P uncaging of caged glutamate.

## Discussion

In this article, we have outlined a cost-effective description of the components necessary for the construction of a one laser source-2P microscope capable of nearly simultaneous 2P uncaging of caged-neurotransmitters and 2P calcium imaging of the activated spines and nearby dendrites. Below we briefly discuss the function of spines that have been elucidated using 2P microscopy, as well as the potential applications of a one-laser system and its limitations.

### Function of Spines

Spines, first described by Cajal (1888) are the main recipient of a neuron’s excitatory input (Gray, 1959; Spacek and Harris, 1998; Arellano et al., 2007). In addition, spines can act as recipients of some GABAergic inputs (Somogyi and Cowey, 1981; Freund et al., 1986; DeFelipe et al., 1989; Chen et al., 2012), and their electrical and biochemical properties are believed to provide them with the capacity to shape how excitatory and inhibitory synaptic inputs are processed and stored (Araya, 2014).

With the development of 2P microscopy (Denk et al., 1990) in combination with 2P uncaging of caged neurotransmitters it has been possible to probe live dendritic spines deep in tissue and with high spatial resolution (Denk et al., 1990; Matsuzaki et al., 2001; Araya et al., 2006b, 2014; Bloodgood and Sabatini, 2007; Harvey and Svoboda, 2007). Using this technology it is well established that dendritic spines are the minimal functional unit for the induction of long-term potentiation (LTP; Lang et al., 2004; Matsuzaki et al., 2004; Harvey and Svoboda, 2007; Tanaka et al., 2008; Araya et al., 2014; Tazerart et al., 2019) and LTD (Holbro et al., 2009; Oh et al., 2013). Furthermore, the development of calcium indicators (Tsien, 1988) in combination with 2P microscopy has allowed researchers to study the spatiotemporal calcium dynamics in dendritic spines during synaptic transmission, LTP and LTD, and synaptic integration (Araya, 2014). In addition, local calcium imaging in activated dendritic spines have allowed us to estimate the effect of spine geometry (i.e., spine head volume and neck length) on the local amplitude and compartmentalization of calcium signals (Noguchi et al., 2005; Sobczyk et al., 2005; Araya et al., 2006b; Grunditz et al., 2008; Takasaki and Sabatini, 2014). These results suggest that spine morphology—spine head volume and/or neck length and diameter—are likely important determinants in controlling the amplitude and diffusion of calcium from the spine head to the dendrite (Araya, 2014). However, the understanding of how spine geometry can affect calcium amplitude and compartmentalization in the spine head has to be understood together with other variables, such as the spine calcium buffering capabilities (Raghuram et al., 2012), extrusion mechanisms (Yuste et al., 2000; Higley and Sabatini, 2012), and the activation of voltage-gated calcium conductances in the spine (Bloodgood and Sabatini, 2007).

Using two-photon (2P) microscopy to image and photo-activate dendritic spines (Denk et al., 1990), we and others have demonstrated experimentally that: (a) spines are electrical compartments (Araya et al., 2006b; Grunditz et al., 2008; Bloodgood et al., 2009; Beaulieu-Laroche and Harnett, 2018) that can attenuate synaptic potentials through the spine neck; and (b) spines are active devices which, upon synaptic activity, can engage voltage-gated Na^+^ (Araya et al., 2007; Bloodgood and Sabatini, 2007; Carter et al., 2012), Ca^2+^ (Bloodgood and Sabatini, 2007), and K^+^ channels (Ngo-Anh et al., 2005; Allen et al., 2011).

The electrical compartmentalization of spines not only affects synaptic transmission but also how inputs are integrated. In fact, it has been shown that nearly simultaneous sub-threshold excitatory inputs onto two or three neighboring spines in basal dendrites of L5 pyramidal neurons summate linearly, whereas neighboring inputs onto the dendritic shaft shunt each other (Araya et al., 2006a). The linear integration of inputs onto spines—before the generation of a dendritic spike—have also been observed when 2P uncaging of caged glutamate was performed in >10 neighboring spines (Gasparini and Magee, 2006; Losonczy and Magee, 2006). Modeling studies have predicted that in order for this linear integration of sub-threshold inputs onto clustered spines to be reproduced, spines with neck resistances of 600 MΩ are required (Grunditz et al., 2008)—similar to the calculated neck resistances in CA1 pyramidal neuron spines (Harnett et al., 2012).

In conclusion, the use of 2P microscopy, together with 2P uncaging of caged glutamate and calcium imaging in the activated spines has allowed us to understand a great deal of the function of spines during synaptic transmission, plasticity and integration of excitatory inputs. We refer the reader to the following review for further information on the function of spines and how this technology has helped uncover the role of spines in input transformations in pyramidal neurons (Araya, 2014).

### A One Laser Source 2P Imaging and Uncaging Microscope

Here, we provide the detailed components required for the construction of a one laser source-2P microscope capable of nearly simultaneous 2P uncaging of neurotransmitters and 2P calcium imaging of the activated spines and nearby dendrites. In particular, we explain the use of an ultrafast tunable Ti:Sapphire pulsed-laser where a single wavelength can be used: (1) at low-laser power on sample, which is not sufficient to result in any uncaging of the caged glutamate compounds, for calcium imaging; and (2) with short high-laser power pulses to 2P uncage caged glutamate. In addition, we describe two types of 2P calcium imaging experiments: linescan (Figures 3A–D) and ROI imaging mode (Figures 3E–H). Linescan imaging mode can be used to obtain a high temporal resolution of calcium imaging within a line crossing the spine head, and ROI imaging mode for experiments where more spatial information (i.e., the calcium signals in the activated spine and its parent dendrite) is required. For details see “Procedure” section.

Although this set-up does allow for the design of sophisticated experiments, it does have some restrictions. First, we are limited in terms of the excitation wavelength of the laser used. Since there is only one laser and the wavelength cannot be rapidly changed from one to another during the experiment, this variable must be set such that it is in the proper range for 2P fluorophore excitation for imaging and for 2P uncaging of caged neurotransmitters and care must be taken to select compatible compounds (see “Materials and Equipments” section). Second, there are also limitations in terms of temporal accuracy. Again, since there is only one laser, uncaging and imaging cannot be performed exactly simultaneously. Specifically, the switch from linescan or ROI calcium imaging to uncaging mode can take ~2 ms. This delay, although not very relevant for the measurement of calcium signals (hundreds of ms), could be detrimental when fast voltage response signals using voltage sensitive dyes (VSD) or genetically encoded voltage indicators (GEVI) are measured in spines (Peterka et al., 2011). A third limitation is the necessity to validate that the imaging laser power is not sufficient to photorelease glutamate, or any other caged neurotransmitter selected, from its cage as demonstrated in Figures 2D,E, and by Tazerart et al. (2019). Finally, another limitation is when multiple spines (>2 or 3 spines) are nearly simultaneously activated while calcium imaging is performed. Under these conditions, the imaging-to-uncaging switch delay is added to the small unavoidable delay between different stimulations, since the pair of galvanometer mirrors directing the laser spot to each spine will have to move from one location to another. To avoid the issue of temporal accuracy, one can combine a conventional galvanometer-based 2P scanning system with a spatially multiplexed imaging/uncaging technique (Nikolenko et al., 2008, 2013). The technique is based on the use of a spatial light modulator (SLM) to generate any desired laser pattern at the sample (Nikolenko et al., 2008, 2013). With the SLM one can split the excitation beam into multiple beamlets and can thus create nearly any spatiotemporal pattern of light, allowing for imaging or photoactivation (uncaging) of multiple regions of interest at once. Hence, with this technology it is possible to simultaneously uncage glutamate (with single spine resolution) at several spines (up to 30 in a 2P regime; Nikolenko et al., 2008, 2013), to study their role in spatial summation. This is a powerful approach, which can be used for true simultaneous activation of a large group of spines.

### Caged Compounds

In recent years, multiple caged compounds have been designed and can be used in conjunction with the techniques described in this article to probe and dissect a variety of brain circuits and function. To be effective, such compounds need to be resistant to spontaneous hydrolysis and to have a rapid photorelease time. For instance, caged glutamate has been one of the most widely used caged neurotransmitters, MNI-glutamate is uncaged in a 2P regime at a wavelength of 720 nm with photorelease half time of ≤0.26 ms (Canepari et al., 2001). In addition to caged glutamate, there is also caged γ-aminobutyric acid (GABA; RuBi-GABA; Rial Verde et al., 2008); 7-(dicarboxymethyl)-aminocoumarin (N-DCAC)—caged GABA; Kantevari et al., 2010), which has been a powerful tool to study for example the role that GABAergic inhibition has on spine function (Chiu et al., 2013).

Furthermore, the development of a novel 2P active caged dopamine compound—RuBi-Dopamine—that can be released with 2P precision in single dendritic spines has been recently described (Araya et al., 2013). Since dysfunction of dopaminergic neurotransmission in the central nervous system (CNS) underlies a variety of neuropsychiatric disorders, caged dopamine allows for the examination of dopaminergic transmission in the brain in both wild-type animals as well as animal models of mental disorders. This compound can further our understanding of neurotransmission at the subcellular level that could potentially be the root of neuropsychiatric disorders. In addition, caged acetylcholine (Passlick et al., 2018), caged glycine (Ellis-Davies, 2007), caged serotonin (Cabrera et al., 2017), caged nicotine (Filevich et al., 2010), and other caged compounds have been developed. We refer the reader to the following reviews for further information on this topic (Ellis-Davies, 2007, 2019).

The single 2P laser approach described here, where a single wavelength is used for 2P imaging spines and 2P uncaging of neurotransmitters, could benefit from the design and development of new caged compounds that could be paired with calcium or voltage indicators. However, one limitation in the design of these novel caged compounds is that, although the absorption and fluorescence spectra can be very well predicted for a one photon (1P) regime using time-dependent density functional theory (TD-DFT; Petroni et al., 2008), the absorption spectra is not accurately predicted for a 2P regime using a similar computational algorithm. This limitation usually makes this endeavor almost purely empirical. Nevertheless, the approach described here could easily be implemented to a battery of existing nitrophenyl-, nitrobenzyl- and ruthenium-based caged neurotransmitters, shown to be successful in probing synapses and neuronal networks in a 2P-regime.

In addition, the experimental approach described here could be used with genetically-encoded calcium indicators (GECIs). Indeed, GECIs provide many advantages over organic calcium indicators, notably avoiding the potential dialysis of intracellular signaling molecules through the whole-cell patch pipette for *in vitro* studies and being more readily usable for *in vivo* studies. However, while the peak 2P absorption of RuBi-caged compounds is compatible with many organic calcium indicators (i.e., Fluo-4, Calcium-green-1, etc.), it is not compatible with currently-available GECI, which have a peak 2P excitation around 880–940 nm (Podor et al., 2015). DEAC450-glutamate is a caged-glutamate compound with peak 2P absorption in the 900 nm range (Olson et al., 2013; Ellis-Davies, 2019). However, this compound emits fluorescence in the 500–540 nm range, making its use with the most commonly-used GECI (GCaMPs) challenging (Ellis-Davies, 2019). Red-shifted GECI could possibly be used with DEAC450-Glu, but they currently suffer from overall poor performance compared with GCaMPs (Podor et al., 2015). Hence, future development of caged-compounds sensitive to wavelengths around 900 nm could extend the applicability of a single-laser source 2P imaging and uncaging system to studies using GECIs *in vitro* or *in vivo*.

## Conclusion

Here, we provide a brief overview on how the use of 2P calcium imaging and 2P glutamate uncaging have helped researchers in the last 15 years unravel the function of spines in: (a) information processing; (b) storage; and (c) integration of excitatory synaptic inputs.

## Data Availability

The raw data supporting the conclusions of this manuscript will be made available by the authors, without undue reservation, to any qualified researcher.

## Ethics Statement

This study was performed in compliance with experimental protocols (13-185, 15-002, 16-011, 17-012, 18-011 and 19-018) approved by the Comité De déontologie de l’Expérimentation sur les Animaux (CDEA) of the University of Montreal, Montreal, QC, Canada.

## Author Contributions

RA conceived the project and supervised the project. DM and ST performed the experiments. DM, ST, and ÉM performed data analyses. RA, ST, and DM designed the experiments. RA, ÉM, and DM wrote the manuscript. All authors read and approved the contents of the manuscript.

## Conflict of Interest Statement

The authors declare that the research was conducted in the absence of any commercial or financial relationships that could be construed as a potential conflict of interest.
